# Supplementary material for: Hemoglobin S and C affect biomechanical membrane properties of P. falciparum-infected erythrocytes
Source: Commun Biol. 2019 Aug 13;2:311. doi: 10.1038/s42003-019-0556-6 (PMC6692299; doi:10.1038/s42003-019-0556-6)
Supplement: Supplementary file 1 — Supplementary Information [file 42003_2019_556_MOESM1_ESM.docx]

**Supplementary Figures**

**
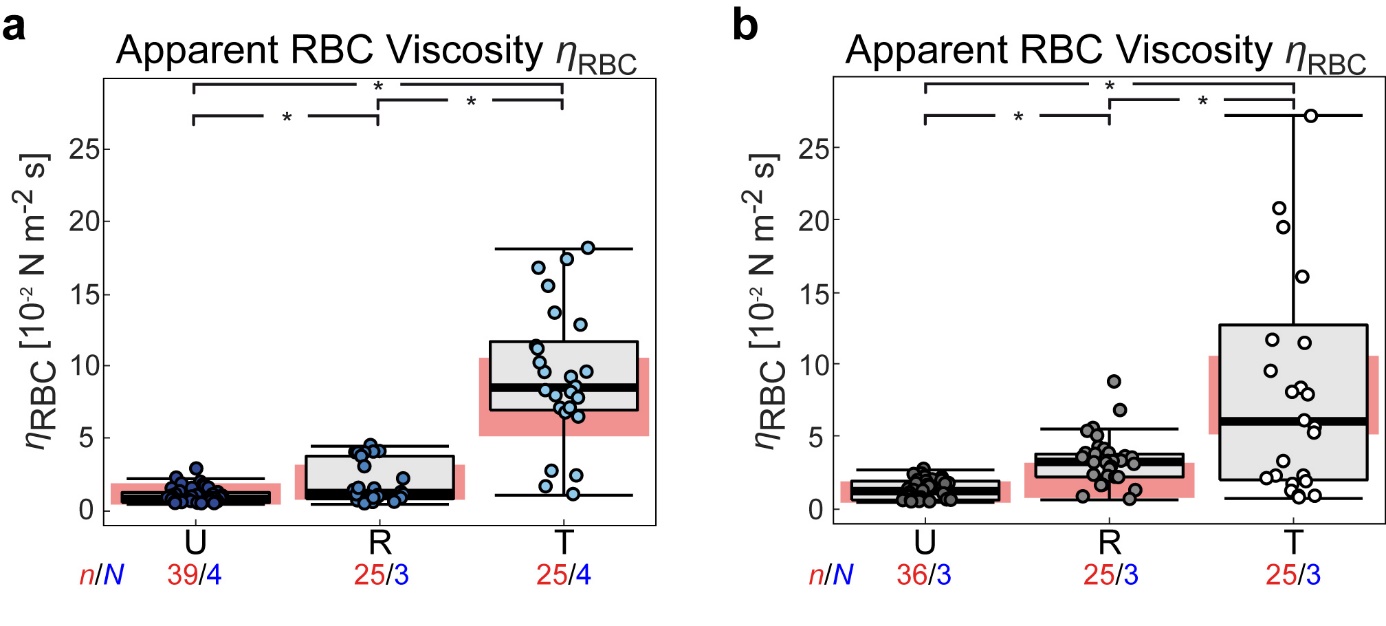
Supplementary Fig. 1** Changes in apparent RBC viscosity in uninfected and *P. falciparum* infected HbAS and HbAC erythrocytes. **a** Apparent red blood cell viscosity *η*_RBC_ for U (dark blue); uninfected, R (blue); ring phase, and T (light blue); trophozoite HbAS erythrocytes. **b** Corresponding values for the apparent red blood cell viscosity *η*_RBC_ for U (dark grey); uninfected, R (grey); ring phase, and T (white); trophozoite HbAC erythrocytes. Individual data points represent a single determination and *n* the total number of data points obtained using blood of *N* different donors. Box plots (grey) were laid over the data points. The 25 – 75 percentile ranges of the corresponding wildtype HbAA results are highlighted in red for direct visual comparison *, *p* < 0.05 according to Welch t-test.

**
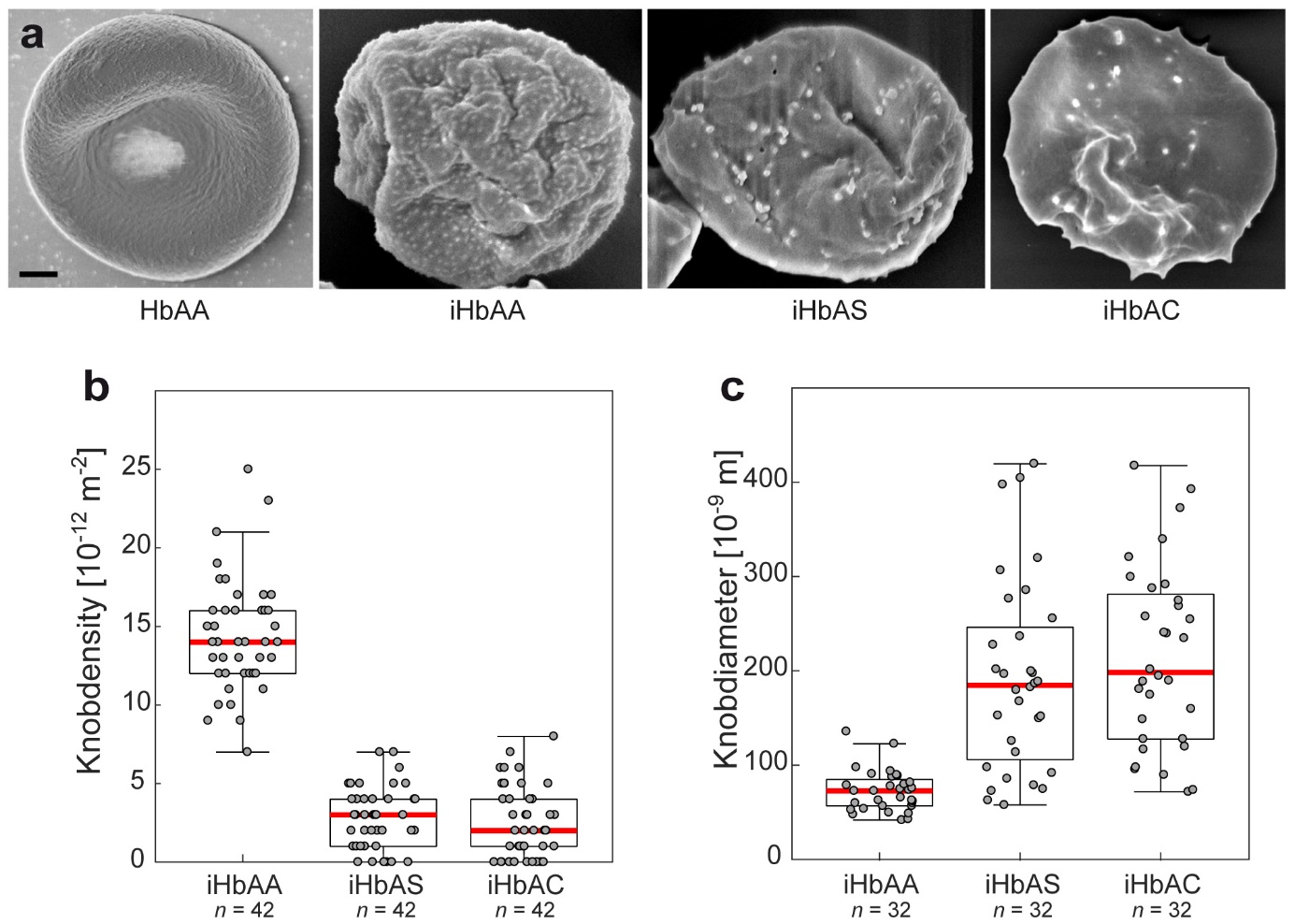
**

**Supplementary Fig. 2** Knob density and knob size. **a** Scanning electron microscopy images of a representative uninfected and infected erythrocytes containing different variants of haemoglobin (trophozoite stage). Scale bar, 1 µm. **b** Knob density and **c** knob diameter, as determined from scanning electron microscopic images. Each data point corresponds to determinations derived from a single cell.


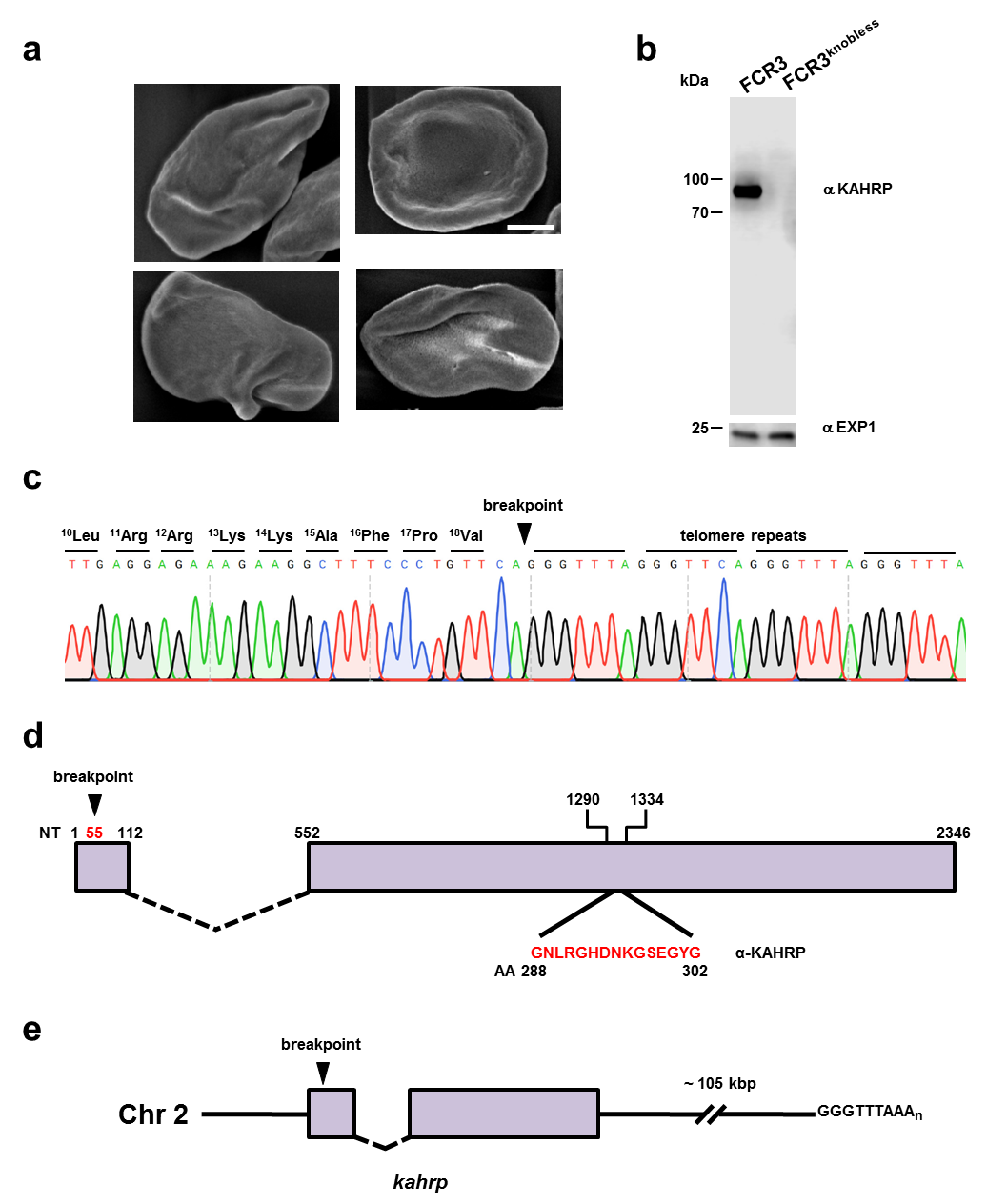


**Supplementary Fig. 3** Molecular characterization of the FCR3 knobless parasite line (FCR3 ^knobless^). **a** SEM images of four representative examples of HbAA erythrocytes infected with FCR3 ^knobless^. **b** Western analysis of total protein extracts from the parental FCR3 line and FCR3 ^knobless^, using an α-KAHRP rabbit antiserum (1:1000) and an α-EXP1 rabbit antiserum (1:1000; provided by Dr. Jude Przyborski). As a secondary antibody a goat α-rabbit antibody conjugated with peroxidase (1:10,000). α-KAHRP antibody was generated using the GNLRGHDNKGSEGYG peptide (AA 288 to 302) (Eurogentec). The last bleeding serum was purified by affinity chromatography. A size marker is indicated in kDa. **c** Chromatogram showing the position of the breakpoint in the *kahrp* gene. To determinate the position of the breakpoint in the *kahrp* gene, PCR reactions were preformed, using the forward primer ATGAAAAGTTTTAAGAACAAAAATAC and a telomere repeat reverse primer (GGGTTTAAA_n_). The resulting DNA fragment of around 0.3 kb was isolated, cloned into the pJET vector (Thermo scientific) and sequenced. **d** Schematic drawing of the *kahrp* gene, including the location of the breakpoint and the sequence used for the α-KAHRP antibody. NT, nucleotide position. AA, amino acid. **e** Schematic drawing of the location of the *kahrp* gene on chromosome 2. The distance between the end of the *kahrp* gene and the telomere repeats are approximately 105 kbp.


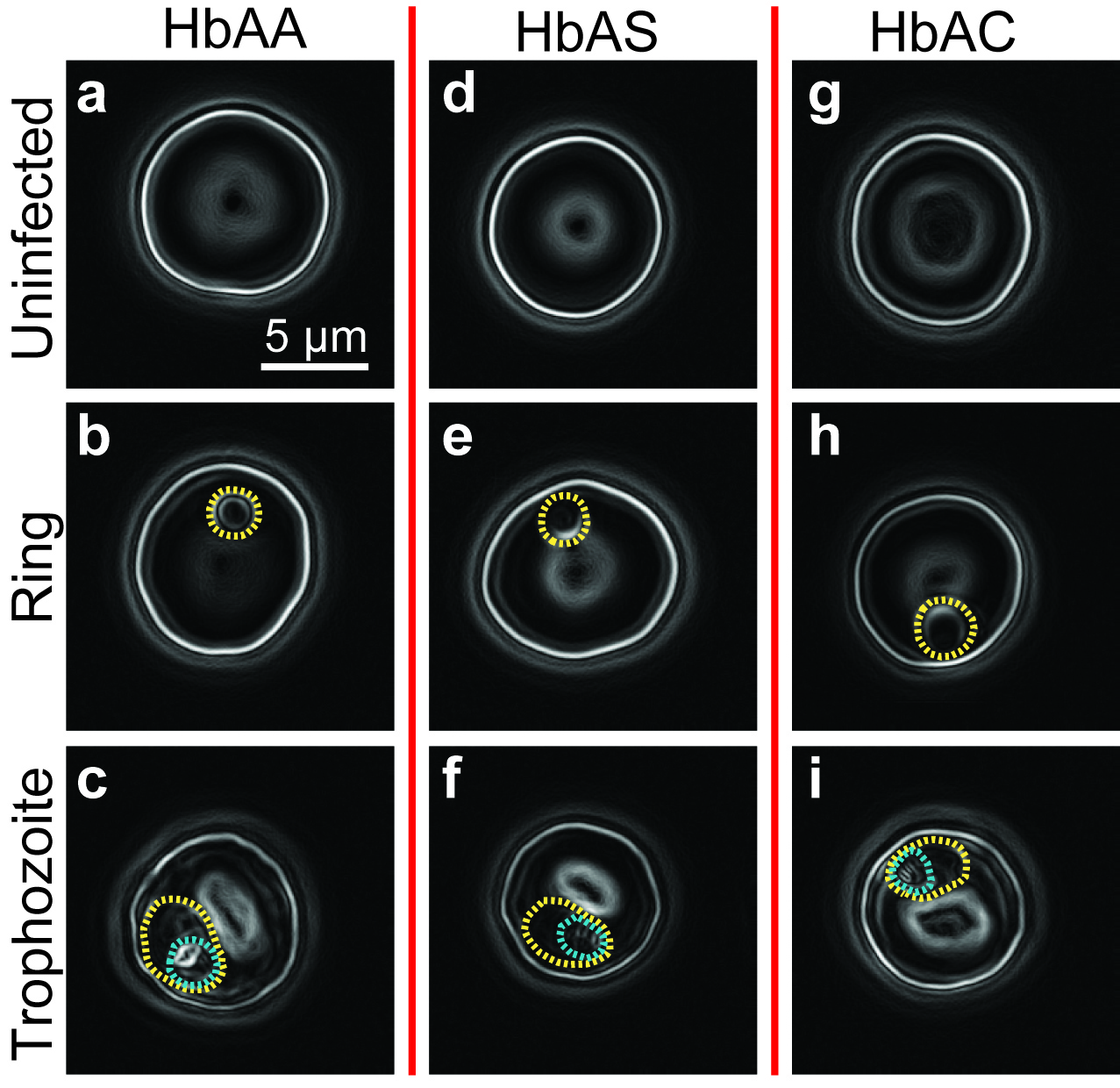


**Supplementary Fig. 4** Gradient images of representative cells of the corresponding parasite stage**.** HbAA (**a-c**), HbAS (**d-f**) and HbAC (**g-i**). The parasite occupied cytoplasmic space is highlighted in yellow, the digestive vacuole, visible at the trophozoite stage, is highlighted in blue. Scale bar, 5 µm

**
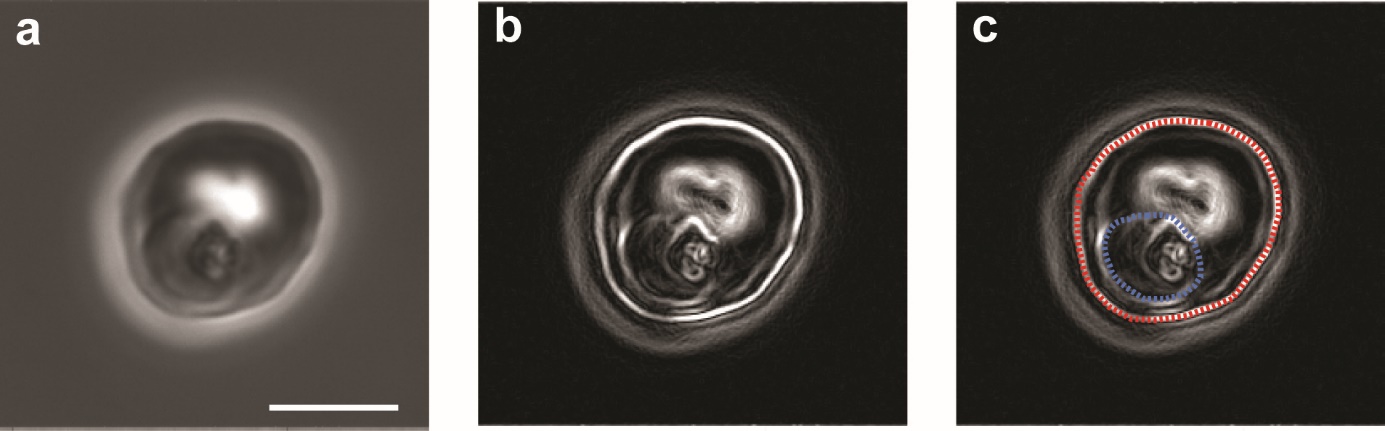
**

**Supplementary Fig. 5** Comparison of phase contrast raw images and derived gradient images. **a** Phase contrast image of an infected erythrocyte (HbAA) at the trophozoite stage and **b** the corresponding gradient image. **c** The gradient image allows for the determination of the cell rim (highlighted in red) and the identification of the parasite-occupied cytoplasmic space (highlighted in blue). Scale bar, 5 µm.

**
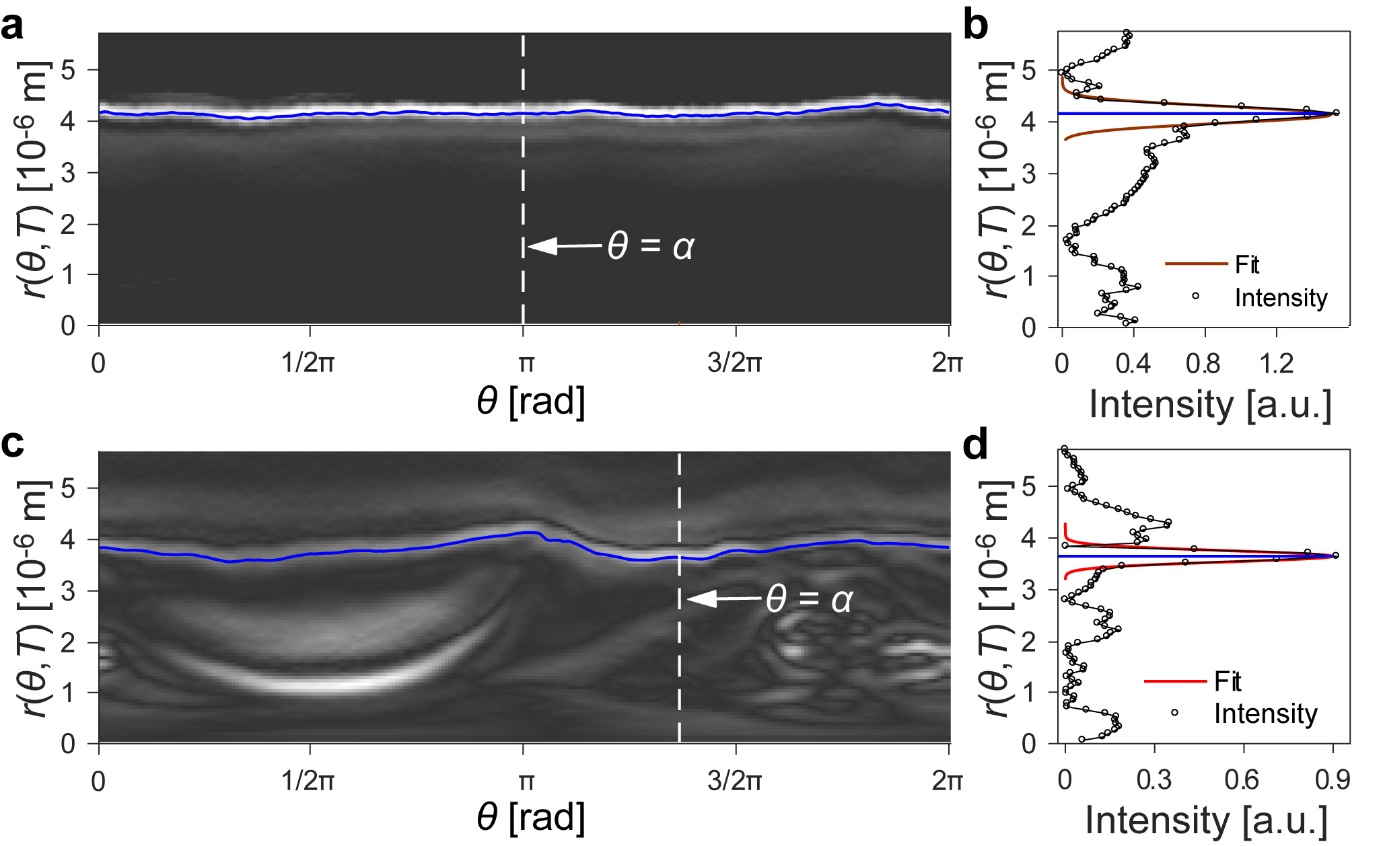
Supplementary Fig. 6** Determination of the cell rim. **a** Gradient map *r*(*θ*,*T*) (HbAA, uninfected) in a polar coordinate for a given time point and the associated cell rim *r*_rim_(*θ*,*T*) of the erythrocyte (blue). **b** As an example, the determination of the rim *r*_rim_(*θ*,*T*) is depicted for the angular position *θ* = *α* (dashed white line in **a**). The intensity maximum was determined from a Gaussian fit. **c**, **d** show the same procedure in case of an infected erythrocyte (HbAA, trophozoite).

**Supplementary Tables**

**Supplementary Table 1.** 2-D Gaussian roughness values obtained from phase contrast images. RMS determined from a Gaussian function fitted to the histograms of the fluctuation amplitude *d* = *r*_rim_(*θ*,*T*) - <*r*_rim_(*θ*,*T* )>*_θ_*_,_*_T_* for each individual cell.

|  |  | Gaussian roughness RMS |
| --- | --- | --- |
|  |  | [nm] |
|  | HbAA | 90 ± 34 (42) |
| Uninfected | HbAS | 94 ± 33 (41) |
|  | HbAC | 103 ± 27 (36) |
|  | HbAA | 137 ± 44 (30) |
| Ring | HbAS | 134 ± 49 (25) |
|  | HbAC | 136 ± 65 (25) |
|  | HbAA | 169 ± 47 (29) |
| Trophozoite | HbAS | 164 ± 77 (39) |
|  | HbAC | 152 ± 55 (40) |
| knobless  Trophozoite | HbAA | 114 ± 31 (47) |

**Supplementary Table 2**. Scaling exponents for different underlying spring network geometries. When varying one parameter, the other one was kept constant at the value indicated in the brackets. The parameter *d*_min_ of the Delauney lattice sets the minimum distance between two junctions and is given in lattice units. Here *d*_min_ = 1 which corresponds to a maximal irregular network.

| Exponent of spring number *N* (*β*) | | Exponent of spring constant *K* (*α*) | |
| --- | --- | --- | --- |
| Square (*k* = 9.2 × 10^-5^ N m^-1^) | 0.9292 | Square (*N* = 400) | 0.9671 |
| Hexagonal (*k* = 9.2 × 10^-5^ N m^-1^) | 0.9552 | Hexagonal (*N* = 423) | 0.9600 |
| Hexagonal (*k* = 9.2 × 10^-8^ N m^-1^) | 0.9480 | Hexagonal (*N* = 379) | 0.9754 |
| Delauney (*k* = 9.2 × 10^-5^ N m^-1^) | 1.0173 | Delauney (*N* = 423) | 0.9447 |

**Supplementary Methods**

**Scaling of confinement parameter with spring number and strength**

In the main text, we vary the spring density and spring constant strength to see its effects on the confinement parameter *γ* and state that the relationship is nearly linear. To find the proper scaling, we define the dimensionless quantities:

| $\Gamma=\frac{\gamma{(L_{x}L_{y})}^{2}}{k_{B}T}, K=\frac{kL_{x}L_{y}}{k_{B}T} \mathrm{and}N,$ | (1) |
| --- | --- |

such that the form of the Hamiltonian suggests:

| $\Gamma\propto K^{\alpha} and \Gamma\propto N^{\beta}.$ | (2) |
| --- | --- |

To find *α* and *β* four our spring lattices, we plot the logarithm of *Γ* against the logarithm of *K* and *N* as it is done for the density and spring number in Figure 6c and d. When varying one parameter, we fix the other one to the value justified in the main text (*k* = 13.2 nN m^-1^) which implies *K* = 3.24 and *N* = 257 for the simulated patch of 1 µm². All other model parameters are kept constant at a value that lies in the RBC relevant range (*κ* = 2.3 × 10^-19^ N m, *σ* = 7.0 × 10^-7^ N m^-1^ and *k*_B_*T* = 4.07 × 10^-21^ N m). The emergent linear relationship implies a good agreement with the scaling properties in Equation 2 and values for α and β are found to be:

| $\alpha=0.9696\pm0.0007 \mathrm{and}\beta=0.9869\pm0.0024.$ | (3) |
| --- | --- |

The exponents are found to be very close to one. In a set of different calculations, we found that the exact value of the exponents is determined by the underlying geometry of the spring lattice. Additional to the hexagonal lattice described in the main text, we employed a square lattice and a Delauney lattice which can have a very irregular spring arrangement. The values for the scaling exponents in each realization are shown in supplementary table 2 and are found to be similarly close to one. This leads to an approximate formula for *γ* depending on the system size, the spring number and the spring strength:

| $\gamma\approx\frac{k_{B}T}{\left( L_{x}L_{y} \right)^{2}}NK.$ | (4) |
| --- | --- |
