# Supplementary material for: Hemoglobin S and C affect biomechanical membrane properties of P. falciparum-infected erythrocytes
Source: Commun Biol. 2019 Aug 13;2:311. doi: 10.1038/s42003-019-0556-6 (PMC6692299; doi:10.1038/s42003-019-0556-6)
Supplement: Supplementary file 2 — Description of Additional Supplementary Files [file 42003_2019_556_MOESM2_ESM.docx]

**Description of Additional Supplementary Files**

**File Name**: Supplementary Data 1

**Description**:  This file contains all underlying raw data as presented in figures 1, 2, 3, 4, 6 and 7 of the main text. Abbreviations and symbols are explained alongside each presented data set.
